# Diagnostic Outcomes of Robotic Bronchoscopy in a Multidisciplinary, Multi-hospital Lung Nodule Program

**DOI:** 10.1016/j.jss.2026.04.031

**Published:** 2026-06-04

**Authors:** Rachel L. Deitz, John P. Ryan, Thomas Rubino, Summer N. Mazur, Corey Kelsom, Omar Awais, Nicholas Baker, Tadeusz D. Witek, Matthew J. Schuchert, Rajeev Dhupar, Stephen P. Kovacs, Ryan M. Levy

**Affiliations:** a Department of Cardiothoracic Surgery, University of Pittsburgh Medical Center, Pittsburgh, Pennsylvania; b Department of Cardiovascular and Thoracic Surgery, West Virginia University School of Medicine, Morgantown, West Virginia; c Department of Cardiothoracic Surgery, Wake Forest University School of Medicine, Medical Center Boulevard, Winston–Salem, North Carolina; d Division of Pulmonary Medicine, University of Pittsburgh Medical Center Comprehensive Lung Center, Erie, Pennsylvania

**Keywords:** Complication rates, Diagnostic yield, Fine needle aspiration, Long-term outcomes, Lung cancer diagnosis, Lung cancer, Robotic bronchoscopy

## Abstract

**Introduction:**

In many centers, robotic bronchoscopy has become the standard approach for diagnosis of lung lesions. Smaller series have demonstrated improvements in diagnostic accuracy. The aim of our study was to assess the diagnostic accuracy of robotic bronchoscopy across multiple users in multiple hospitals within a single health system with the largest patient cohort in the literature to date.

**Methods:**

All patients with a suspicious intraparenchymal lung lesion that was biopsied using the Monarch robotic bronchoscope by a pulmonologist or thoracic surgeon at a multi-site, academic institution from August 2018 to April 2023 were included. Data were collected prospectively, and patients were followed postprocedure from at least 1 and up to 5 y. Diagnostic accuracy was determined by comparing Monarch-acquired samples to final known pathology. True negative (benign) diagnoses were confirmed with either resolution or stability on computed tomography scan at one-year post-procedure.

**Results:**

Over the five-year period, 1084 lesions were biopsied via robotic bronchoscopy (Monarch) by 16 practitioners. Lesions were primarily solid (84%) and peripherally located (62.0%), with a median size at 25 mm (17–37). At median follow-up of 1.07 (interquartile range 0.58–1.67) years, diagnostic accuracy was 82.7% (80.1–85.2). In univariate analysis, larger lesion size (*P* < 0.001), peripheral location (*P* < 0.001), bronchus sign on computed tomography scan (*P* < 0.001), intraoperative direct endobronchial visualization (*P* < 0.001), and use of radial endobronchial ultrasound (*P* < 0.001) were all associated with successful diagnosis. Clinically significant pneumothoraces (requiring chest tube) were low at 3% (*n* = 33).

**Conclusions:**

Diagnostic accuracy from this large cohort, cross-specialty analysis of robotic bronchoscopy among multiple operators was 82.7%. Robotic bronchoscopy can be successfully employed as the initial and preferred sampling methodology for solid lung lesions given acceptable diagnostic performance and low complication rates in this long-term assessment of outcomes.

## Introduction

Lung cancer remains the third most common cancer in the United States. It is the leading cause of cancer-related deaths, with approximately 125,070 deaths attributable to lung cancer in 2024.^1^ For tissue diagnosis of pulmonary lesions, the gold standard tool has traditionally been computed tomography–guided fine needle aspiration (CT-FNA) which has accuracy approaching 94%, but rates of pneumothorax development in as high as one in four cases.^2^ An endobronchial approach for the sampling of pulmonary lesions, such as electromagnetic-guided navigational bronchoscopy or thin bronchoscopy with radial endobronchial ultrasound (radial EBUS), confers a significant reduction in risk of pneumothorax but has a tradeoff in reduced diagnostic accuracy of around 70%.^3,4^

More recently, three platforms using robotic bronchoscopy have been adopted in which an endobronchial approach is coupled with advanced navigation techniques to improve diagnostic accuracy^5^ and reduce rates of pneumothorax.^6^ Yield rates in smaller studies evaluating these platforms are high but are subject to several caveats such as small sample size,^7^ use of adjunct imaging modalities to improve accuracy,^8^ or operation by a single or few diagnosticians^9^ which limit their generalizability.

Our institution began to utilize the robotic bronchoscopic platform shortly after its introduction and participated in early multicenter reporting of initial safety, feasibility, and diagnostic yield rates.^10^ This platform was rapidly adopted across our institution, spanning multiple users, departments, and sites (across >130 miles), enabling long-term follow-up and evaluation of its diagnostic success. The objective of the present study is to provide a large, real-world examination of the safety and utility of robotic bronchoscopy in the diagnosis of pulmonary lesions and provide guidance on the characteristics of nodules best suited for this approach.

## Methods

### Patient selection

All patients who underwent robotic bronchoscopy for the tissue diagnosis of a suspicious lung lesion at four hospitals were included in this study. Encounters between August 1, 2018, and April 30, 2023, were included in the study. Study data were collected and managed using REDCap electronic data capture tools hosted at The University of Pittsburgh.^11,12^ Basic demographic information, health history, imaging studies, operative data, and postoperative sequelae were captured. The study was approved by the University of Pittsburgh Human Research Protections Office (study #2104–0181), which included a waiver of informed consent.

### Robotic bronchoscopic technique

Robotic bronchoscopy was performed using the Monarch (Johnson and Johnson, New Brunswick, NJ) robotic bronchoscope in the standard fashion according to the manufacturer’s guidelines and as described previously.^13^ All operations occurred under general anesthesia with orotracheal intubation. Navigation was performed by the proceduralist (either pulmonologist or thoracic surgeon) to the target lesion or lesions of interest and biopsied with a combination of needle (18 gauge-21 gauge) and/or forceps. Use of image enhancement via operating table two-dimensional fluoroscopy or radial EBUS probe to help visualize the lesion was left up to the discretion of the proceduralist. No other adjunct imaging modalities (i.e., cone beam CT) were utilized. Decisions regarding the number of specimens or additional procedures performed at the time of the operation were individualized to the patient and left to the discretion of the proceduralist.

### Lesion characteristics

Lesion characteristics such as nodule size, density, and location were extracted from preprocedural CT scans that were obtained less than 30 d prior to the robotic bronchoscopy. Determination of the presence of bronchus sign (visible bronchus leading to lesion) was determined in 0.6-mm fine cuts in either axial or coronal views. Lesion centrality was defined as a lesion within 20 mm of the hilum or lobar bronchus. Lesion data were noted by the radiologist and independently confirmed and reviewed for accuracy by a second radiologist and a thoracic surgeon. Follow-up CT scans to evaluate for lesion growth, stability, or regression were similarly independently reviewed. Specimens were examined by cytopathology departments individual to each hospital where the biopsy was performed. Rapid on-site analysis was used at the discretion of the operator if available at the site where the procedure was performed.

### Determination of accuracy

Diagnostic accuracy was calculated in concordance with previously published methodology in determining the diagnostic success of samples obtained via bronchoscopy.^14,15^ Samples were considered diagnostic if malignant tissue confirmed by histopathological analysis was obtained. Benign samples were considered diagnostic provided either (a) an additional biopsy was performed on the index lesion that failed to detect malignancy or (b) serial CT imaging demonstrated lesion stability a minimum of six months post-procedure. A subgroup analysis was conducted using one-year follow-up as the time point for stability. Atypical samples were considered nondiagnostic. Samples were also considered nondiagnostic if malignancy was not detected in a lesion, but the lesion was suspicious enough to warrant targeted treatment, such as stereotactic body radiotherapy (SBRT). Lesions from patients who had incomplete follow-up (no repeat scan to evaluate stability) and lesions from patients who had chemotherapy initiated in the setting of nonrelated cancer diagnosis within six months postprocedure were excluded from the final analysis for diagnostic accuracy.

### Statistical analysis

Univariable analyses were performed to compare preoperative and postoperative characteristics between patients based on diagnostic success. Categorical variables were analyzed with Fisher’s exact test, and continuous variables were analyzed with Wilcoxon rank sum test. Post hoc comparisons of categorical variables with more than two levels were performed by examining the adjusted standardized residual from a chi-square test. Rates of diagnostic accuracy were calculated with a 95% Wald confidence interval (CI). All analyses were performed in R (version 4.5.2). Univariable analyses were performed using the gtsummary^16^ package. A multivariable regression analysis was performed to identify significant predictors of successful Monarch diagnosis among the patient, operative, and nodule variables. To optimize the model, we employed a bidirectional stepwise selection procedure based on the Bayesian Information Criterion (BIC). The BIC was chosen as the criterion for model selection due to its effectiveness in penalizing model complexity, thus helping to prevent overfitting. Model fit was assessed using the Hosmer–Lemeshow test. To estimate the procedural learning curve for achieving Monarch success, we calculated the cumulative success rate for each operator as the cumulative number of successful cases divided by the cumulative number of procedures performed up to that case sequence. The mean cumulative success rate across operators was then plotted against case sequence number to visualize overall trends in performance improvement. To evaluate the impact of early surgeon experience, we conducted sensitivity analyses excluding the first 5, 10, and 15 cases for each surgeon. The association between cumulative surgeon experience and procedure time was assessed using a linear mixed-effects model, with case sequence number as a fixed effect and surgeon included as a random intercept to account for within-surgeon correlation. Analyses were restricted to surgeons performing at least 20 procedures. Values are reported as medians (interquartile range) unless otherwise indicated. A *P* value of <0.05 was considered statistically significant for all analyses.

## Results

### Case experience

Between August 2018 and April 2023, 1084 lesions were biopsied during 1022 procedural encounters. Biopsies were performed by 16 different operators which included seven pulmonologists and nine thoracic surgeons. Operative data are displayed in Table 1. More cases were performed by a pulmonologist (*n* = 690, 67.5%) than by a thoracic surgeon. Median case time was 58 (46–73) min, during which two-dimensional fluoroscopy was used for image-guided enhancement in 96.6% of cases (*n* = 986). Traditional linear EBUS for concomitant lymph node evaluation was performed at the time of the index operation and 53.5% (*n* = 547) of the time. Additional procedures were performed in 48 cases, most commonly esophagogastroduodenoscopy (*n* = 25, 2.4%) followed by chemotherapy port placement (*n* = 10, 1.0%) or fiducial marker placement (*n*-6, 0.6%).

### Patient data

Demographic patient data are reported in Table 2. The cohort was predominantly White (92.5%, *n* = 945) with slightly more female patients (53.4%, *n* = 546). The median patient age was 68.9 (62.9–75.58) y. Most patients were current (*n* = 364, 35.6%) or former (527, *n* = 51.6%) smokers. More than half of the patients had a history of chronic obstructive pulmonary disease (COPD, *n* = 537, 52.5%). Almost one-third had a history of nonpulmonary cancer (*n* = 322, 31.5%) and 11.1% (*n* = 113) had a history of lung cancer.

### Lesion characteristics

Nodule characteristics are reported in Supplemental Table 1. The majority of the biopsied lesions were located in the right lung (*n* = 627, 58%), with an overall predominance of bilateral upper lobe lesions (*n* = 600, 55.4%). The median long axis was 25 (17–37) mm, and the short axis was 18 (13–27) mm. Most were located in the periphery beyond the hilum or lobar bronchus (*n* = 667, 62%). Solid nodules were the most common (*n* = 901, 84%), and most lesions had a visible bronchus sign (*n* = 760, 70%) on CT. The median follow-up time for serial CT imaging of the lesions was 1.07 (0.58–1.67) years.

An additional 29 lesions (2.6% of all lesions) were attempted to be biopsied but could not be reached by the robotic system. Patient and operative characteristics between lesions that were reached and those not reached are displayed in Supplemental Table 2. Lesions that were unable to attain adequate navigation were smaller (long axis: 16^6,13–26^ *versus* 25 [17–37] mm, *P* = 0.002; short axis: 13^10–19^ *versus* 18^6,13–26^ mm, *P* < 0.001) and less likely to display a bronchus sign on CT (41% *versus* 71%, *n* = 12 *versus* 760, *P* < 0.001). There were no patient characteristics that differentiated whether a lesion could be reached. The greatest proportion of lesions unreachable by Monarch was found in the right upper lobe (*n* = 13, 45%), with 28% of the unreachable lesions (*n* = 8) residing in the apical segment, though the comparison between lung, lobe, or segment did not reach statistical significance.

### Complications

Postoperative respiratory failure occurred in eight patients (0.8%). In an additional seven patients (0.7%), a new supplemental oxygen requirement was detected postoperatively, requiring admission for home oxygen evaluation and therapy coordination (Supplemental Table 3). Three patients (0.3%) remained intubated postoperatively for endobronchial bleeding, all of whom were extubated the following day after toilet bronchoscopy. The most common complication, pneumothorax, occurred in 51 patients (5.0%), including 33 cases in which tube thoracostomy was performed (3.0% of all cases). There was no temporal association with rates of pneumothorax across the study period. There were no associations between patient characteristics, including history of COPD or history of chest surgery, and increased risk of requiring tube thoracostomy (Supplemental Table 4). Use of radial EBUS probe during the procedure was associated with a lower risk of significant pneumothorax (*P* = 0.012). The combined bilateral upper lobes represented 42% of pneumothoraces requiring tube thoracostomy (right upper, *n* = 7; left upper, *n* = 7). There were differences in the risk of requiring tube thoracostomy depending on the lung segment biopsied that did not reach statistical significance (*P* = 0.063). More chest tubes were required after biopsy of right middle medial segment (*n* = 4, 12.0%) and the left upper apicoposterior segment (*n* = 4, 12.0%) than in other segments.

### Diagnostic accuracy

**Fig. 1 –:**
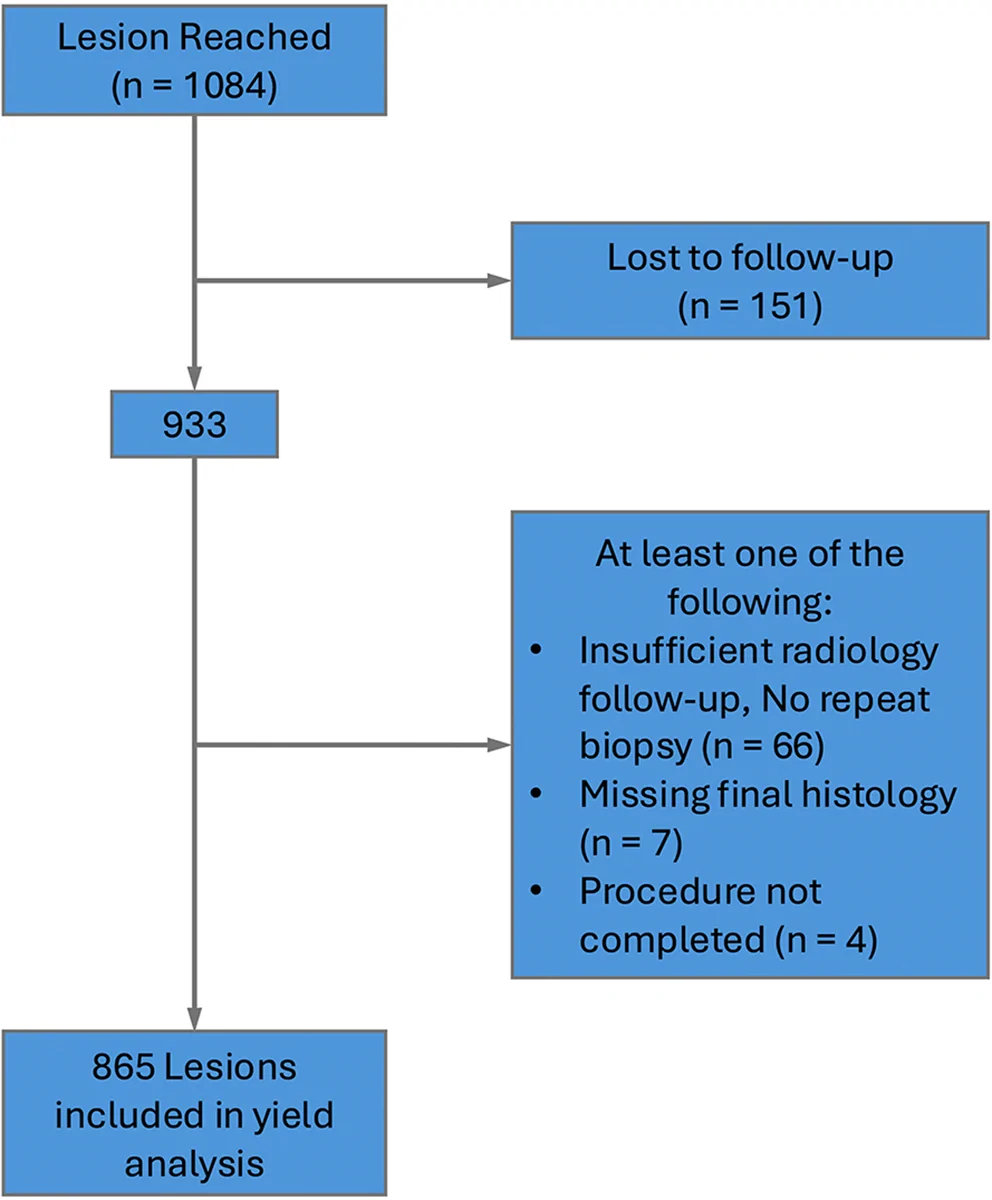
Consort of nodules included in yield analysis.

A total of 865 lesions (Fig. 1) were included in the analysis for diagnostic accuracy (Table 3). Overall diagnostic accuracy in the cohort was 82.7% (95% CI: 80.1–85.2). When either the first 10 cases performed by each surgeon or when the first 10 cases performed at each hospital were eliminated from the analysis, the rates remained consistent at 82.2% (95% CI: 79.3–85.0). Success rates were nearly identical when excluding the first five (82.0%), 10 (82.2%), or 15 (82.4%) cases, with overlapping 95% CIs, indicating no evidence of a cutpoint effect. When lesions with less than 1 y of imaging follow-up were eliminated from the analysis, there was no significant change in rates of diagnostic accuracy (82.2%, [95% CI: 79.7%-84.8%]).

### Correlates of Monarch success

Age, race, smoking status, and history of lung disease were not associated with successful diagnosis (Table 3). Success was not associated with procedure time or development of minor complications. There was a laterality trend with tumors in the right lung less likely to be successfully diagnosed (55% *versus* 45% in left, *P* = 0.053). There were no significant differences in success rates when stratified by lobe (*P* = 0.28) or segment (*P* = 0.69).

Nodule characteristics were strongly associated with diagnostic success. Lesions successfully diagnosed with the robotic bronchoscope had greater long (27 [18–40] mm *versus* 21 [15–31] mm, *P* < 0.001) and short (20 [14–30] *versus* 15,^6,12–21^ *P* < 0.001) axis lengths and shorter distance from the pleura (6 [0–19] mm *versus* 8 [0–21] mm, *P* = 0.018). Central lesions were associated with lower rates of Monarch success (42% *versus* 58% peripheral, *P* < 0.001), while the presence of bronchus sign was associated with greater odds of successful diagnosis (74%, *P* < 0.001). The overall yield rate of lesions with bronchus sign on CT was 85.5%.

Intraoperative characteristics were also associated with diagnostic success. Lesions visible in the periphery at the tip of the bronchoscope were more likely to be successfully diagnosed than those that were not visible (25% *versus* 15%, *P* = 0.002), and successfully diagnosed lesions were also more likely to have used radial EBUS to confirm targeting of lesion prior to biopsy (72% *versus* 52% that were not successfully diagnosed, *P* < 0.001).

**Fig. 2 –:**
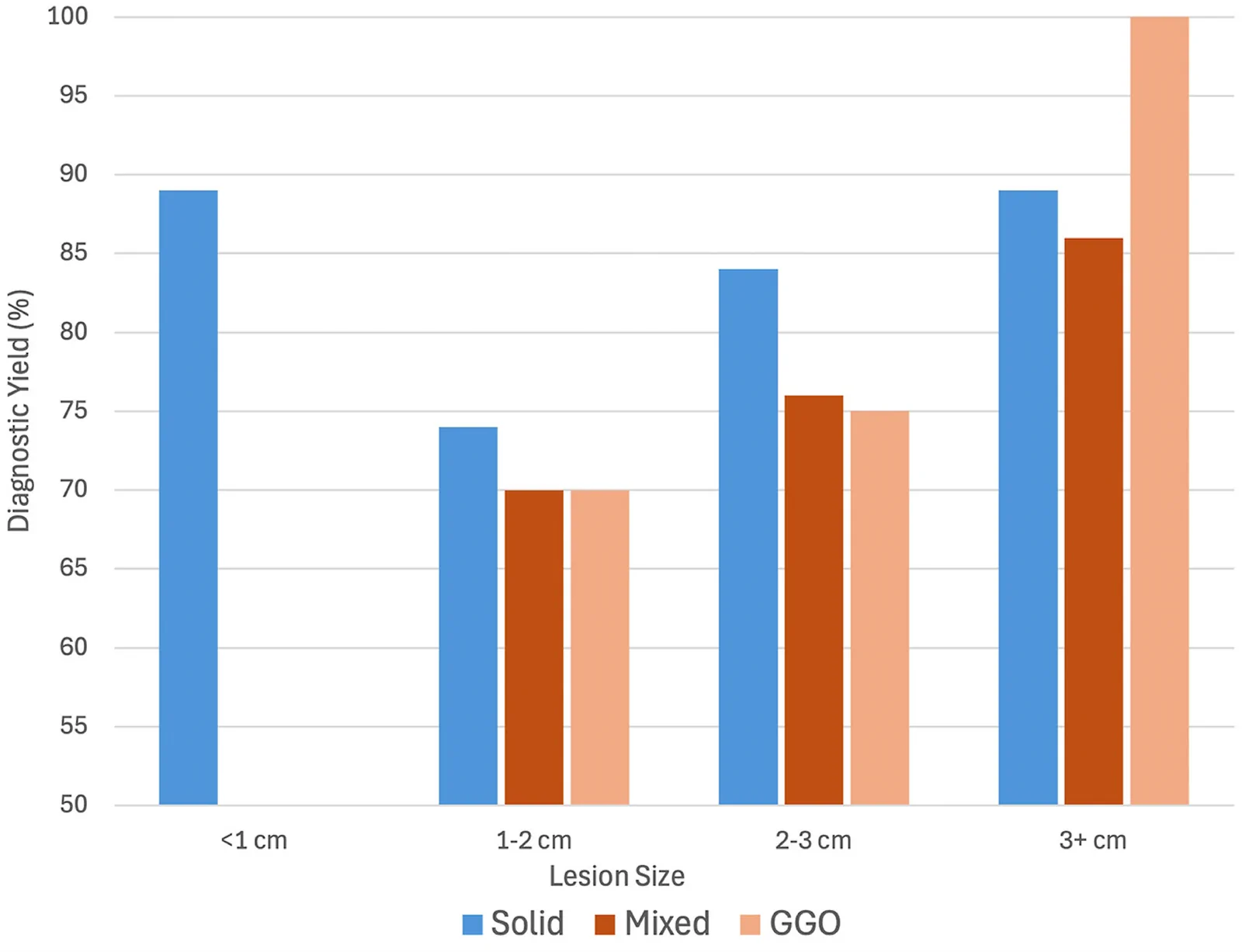
Diagnostic accuracy stratified by lesion size and character.

Rates of accuracy for lesions stratified by size and density on cross-sectional imaging are reported in Table 4. The diagnostic accuracy from lesions between 1 cm and 2 cm was 74%. A combined rate of 86.3% was obtained in the biopsy of all lesions of >2 cm, with the highest overall rate of 89% obtained from solid lesions of >3 cm. Small ground glass opacities or mixed density lesions of less than 2 cm had the lowest rates of diagnostic success (70%), but the overall number in these subgroups (*n* = 33 lesions) was small (Fig. 2).

**Fig. 3 –:**
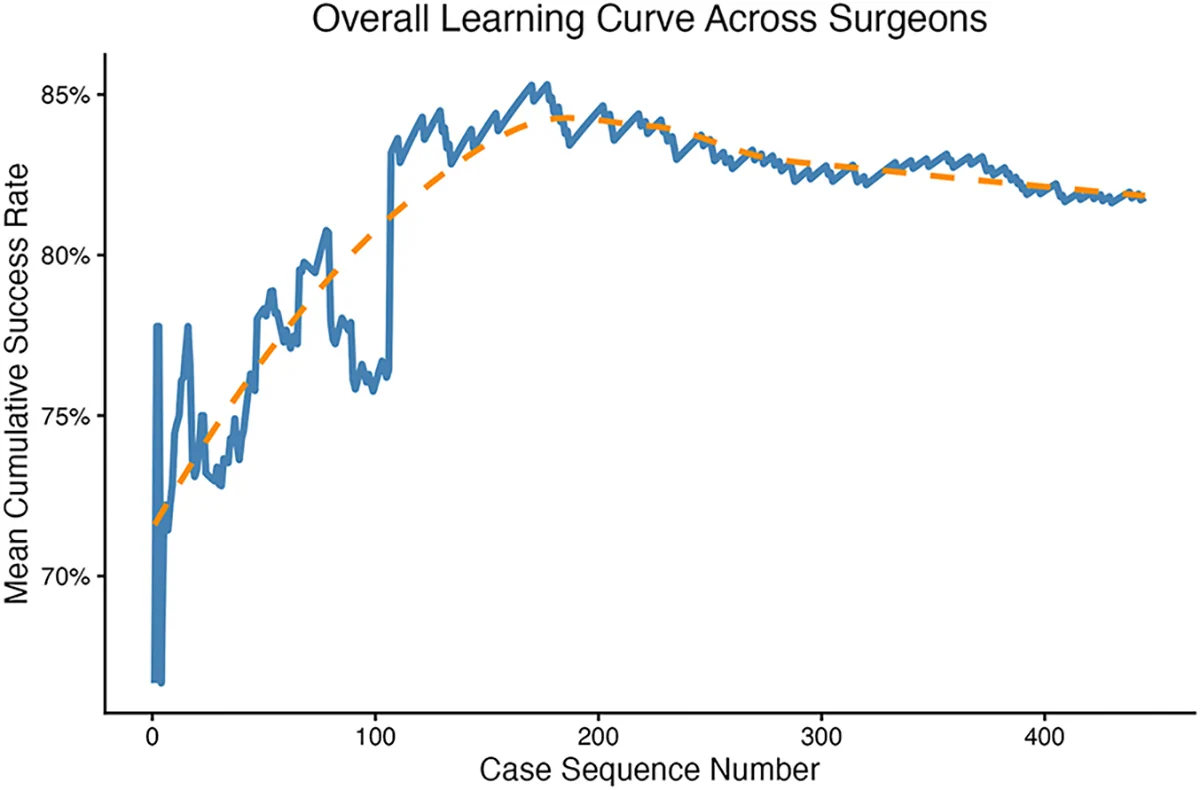
Mean cumulative success by case number.

Operator experience, as quantified by case number, was associated with an increased rate of diagnostic success. Operator success increased consistently over the first 130 cases from a baseline rate of approximately 72% to a plateau of approximately 84% (Fig. 3). Procedure time decreased significantly with increasing surgeon experience (−0.041 min per case; *P* < 0.001). This corresponds to an average reduction of approximately 4 min over the first 100 cases. Procedure time varied substantially between surgeons (standard deviation 10.2 min), even after adjustment for cumulative case number.

### Multivariable analysis

**Fig. 4 –:**
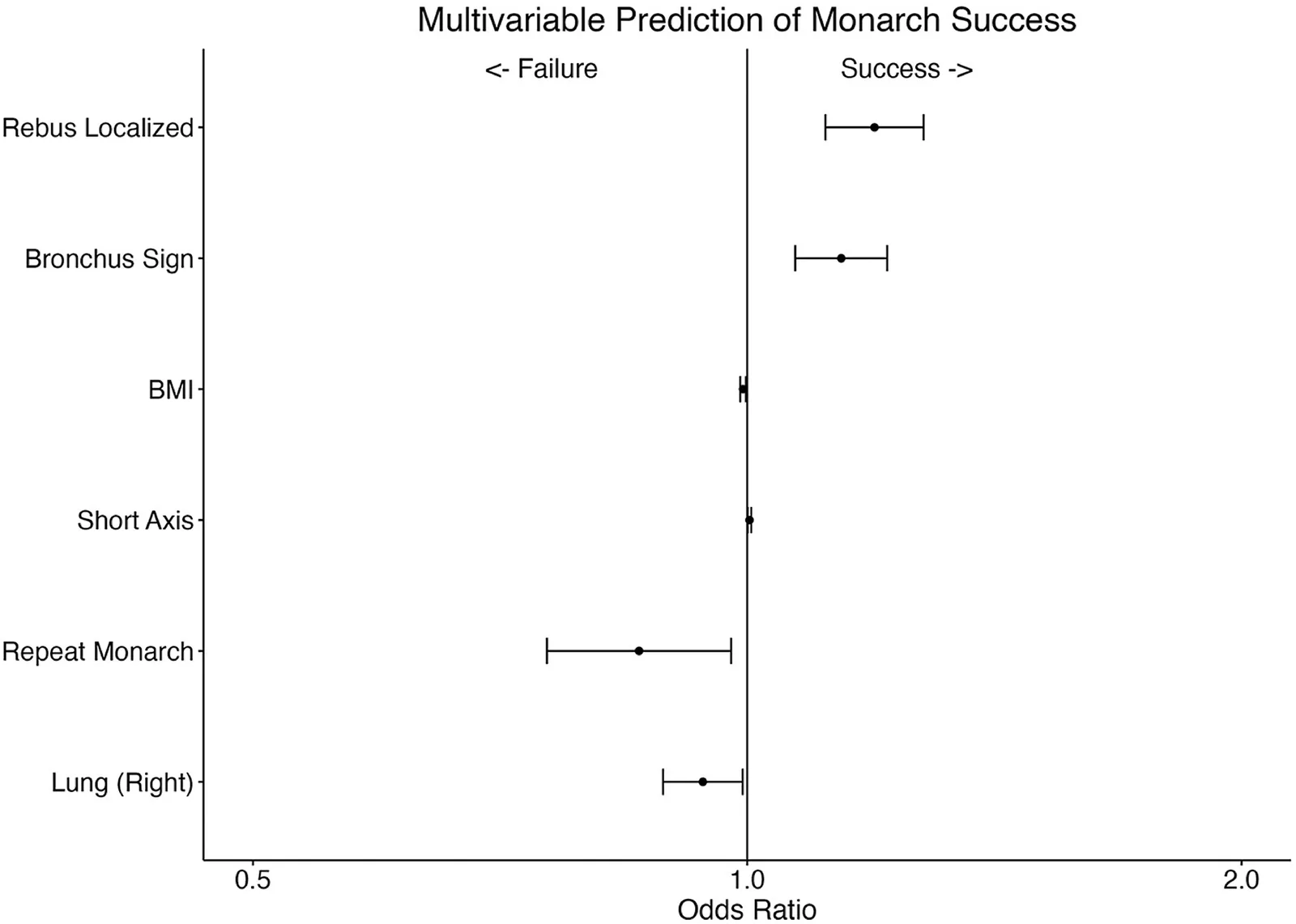
Multivariable prediction of Monarch success.

Stepwise feature selection identified six variables that were the optimal model for predicting Monarch success (Table 5, Fig. 4). The final model displayed acceptable fit (χ^2^[8] = 19.67, *P* = 0.01) and had a BIC of 710.3. Higher body mass index (odds ratio [OR] = 0.99 [95% CI: 0.99–1.00], *P* = 0.003) and repeat Monarch (OR 0.86 [95% CI: 0.76–0.98], *P* = 0.022) were associated with lower likelihood of Monarch success. Lesions on the right side had a lower probability of successful diagnosis (OR 0.94 [95% CI: 0.89–0.99], *P* = 0.017). Positive bronchus sign (OR 1.14 [1.07–1.22], *P* < 0.001) and radial EBUS localization (OR 1.20 [1.12–1.28], *P* < 0.001) were both associated with better likelihood of success.

### Indeterminate lesions

An indeterminate result that yielded cellular atypia of uncertain significance was obtained from 9.4% of cases (*n* = 102, Table 6). Compared to a benign or malignant diagnosis, these lesions were less often found in never smokers (*P* < 0.001) and more often in former or current smokers, in patients with a history of COPD (*n* = 70, 69%, *P* = 0.006), and in those with a higher Charlson Comorbidity Index. Indeterminate lesions were smaller, (median long axis of 23 [16–36] mm), more often peripheral (*n* = 68, 67%), and more likely to have mixed density attenuation (*n* = 20, 20%). They were most often obtained from within the right upper lobe (*n* = 40, 39%), specifically in the apical segment (*n* = 26, 25%, *P* = 0.003). Postprocedure, 41.2% (*n* = 42) of these indeterminate lesions were monitored, with 58.8% (*n* = 60) referred for resection (*n* = 24) or rebiopsy (*n* = 36, Supplemental Fig. 1). Malignancy was identified in 81.7% of these cases (*n* = 49). A further 11 cases were referred for empiric SBRT due to high suspicion of malignancy.

### Tumor malignancies

A malignant diagnosis was obtained by the robotic bronchoscope in 470 lesions with an overall sensitivity for detection of malignancy at 71.8% (Supplemental Table 5). The remaining 185 malignant nodules not diagnosed by robotic bronchoscopy required either repeat robotic bronchoscopy, CT-FNA or surgical resection for diagnosis. Adenocarcinoma was the most commonly detected histologic subtype (*n* = 212), followed by squamous cell carcinoma (*n* = 134) and metastasized cancer (*n* = 34). Surgical resections were performed in 261 cases in which the predominant tumor stage resected was a T1b lesion. SBRT was performed in 121 cases, and microwave or cryoablation was performed in four cases.

## Discussion

Improving survival in patients with lung cancer is a key national public health objective. A significant survival benefit is conferred upon eligible individuals who undergo lung cancer screening with low-dose CT, as demonstrated in both the US National Lung Screening Trial and Dutch-Belgian lung cancer screening (NELSON) trials.^7,18^ Expanded screening, however, is intimately associated with the potential harms of workup or surveillance, including the sequelae of invasive diagnostics.^19^ Despite the high diagnostic accuracy of CT-guided FNA, more than one quarter of patients may suffer a pneumothorax,^2^ with several studies noting complication rates approaching 40%.^20^ This potential complication may delay prompt oncologic treatment or diagnosis or deter patients from completing a diagnostic workup.

The objective of our study was to compile a large cohort of patient and operative data to assess diagnostic accuracy with robotic bronchoscopy in a multiuser, multi-hospital single health system. In our experience of over 1000 cases, rates of pneumothorax were very low (3.0% in our series) compared to historical rates of pneumothorax conferred by use of CT-guided FNA. Our diagnostic success rates were high at 82.6%, slightly higher than the pooled yield of a meta-analysis by Nadig *et al*. ^21^ that included 483 nodules. Our analysis demonstrated even greater diagnostic success in lesions that were selected for size, character, and additional imaging characteristics (such as CT bronchus sign). The use of radial EBUS probe was also strongly associated with diagnostic success in our series. While similar predictors of success were found by Murgu *et al*. ^6^ we were able to report on a larger series with additional granularity on predictors for success, pneumothorax, and indeterminate findings. We were additionally able to characterize anatomic lung segments that may be associated with poorer diagnostic accuracy, rates of pneumothorax, and indeterminate results, allowing providers to better predict case outcomes and select patients for robotic bronchoscopy. We observed that right upper lobe lesions may provide the greatest challenge to operators, as they represented the greatest proportion of lesions that were either unable to be biopsied or to yield an indeterminate or inaccurate result. Biopsies of these apical lesions, particularly in cases where the results are indeterminate, should be followed closely and considered for repeat bronchoscopic or excisional biopsy. Other patient characteristics, such as increased body mass index, were associated with increased odds of unsuccessful diagnosis. Additional examination of patient-level characteristics may be helpful to determine whether changes to intraoperative strategies, preoperative imaging, or selection may help optimize diagnostic accuracy in various subgroups.

For sampling other challenging lesions, such as small, peripheral sub-solid nodules, or pure ground glass opacities, augmented imaging with either cone beam CT or a tomosynthesis system with augmented fluoroscopy^22^ may be an important adjunct to improving diagnostic accuracy. The combination of robotic bronchoscopy and cone beam CT guidance can produce very high diagnostic yield rates^23^ with a trade-off in high resource utilization and lengthy procedure times^24^ that may not be necessary in most cases. For cases that are appropriate to proceed to resectional biopsy, intraoperative localization of sub-centimeter nodules and ground glass opacities can be similarly difficult, and we have had good success with both preoperative bronchoscopic dye marking^25^ and intraoperative localization with fluorescence after systemic injection of pafolacianine.^26^

In contrast to diagnostic yield calculations determined at the time of biopsy, as described by Gonzalez,^27^ we conducted a retrospective assessment of diagnostic accuracy based on long-term data within our large cohort. This approach allowed us to quantify the proportion of patients whose nodules resolved, indicating a true benign diagnosis, or progressed or required rebiopsy, indicating a true malignant diagnosis. Reporting outcomes based on long-term follow-up, such as was used in the VERITAS trial,^15^ may more closely mirror real-world decision-making regarding the follow-up of “benign” lesions and the selection of cases for repeat biopsy.

Our series was also unique in that we included multiple operators from different specialties and different practice patterns. While smaller series may focus on procedural outcomes for a small number of high-volume operators, this series included both pulmonologists and surgeons who were introducing this procedure into their practices and beginning to accumulate case volume. Though cumulative success of this procedure increased over time based on case experience, even the initial case experience provided adequate diagnostic accuracy comparable to other published data. These proceduralists were able to easily adopt this new technology without detriment to outcomes. Procedure time also decreased with increasing surgeon experience, though a 4-min reduction may not have had substantial impact on daily workflow. After adjusting for case number, procedure time remained variable between operators, suggesting that factors beyond experience alone, such as procedural complexity, addition of adjunct cases, and other intraoperative factors, may meaningfully influence procedural times. Lastly, while the use of radial EBUS during the procedure conferred an advantage to successful diagnosis, enhanced imaging techniques such as cone beam CT were not employed. Thus, our results are more likely to be generalizable to adoption of robotic bronchoscopy in a variety of settings.

Some limitations should be noted that are inherent to the design of the study. First, the decision to pursue robotic bronchoscopy was left to the discretion of the physician; thus, a selection bias is likely present with regard to characteristic lesions that may have been chosen for this approach. Second, confirmatory pathologic diagnoses were only made in patients who underwent lung resection, and the true proportion of patients with a false negative/benign diagnosis cannot be known with certainty. A subset of patients was lost to follow-up, died, or declined to get repeat imaging, introducing potential bias into the cohort.

Despite these limitations, our study in its large sample size and number of users allow for greater generalizability and confirmation of certain findings that have been published in smaller series, such as the use of bronchus sign as a predictor of success^28^ and the higher rates of diagnostic failure in smaller lesions.^21^ While sampling of larger, solid lesions yielded high success rates, the second most frequently biopsied lesion in our cohort was between 1 cm and 2 cm and led to a successful diagnosis in only 74% of cases. With our aim to detect lung cancer in patients at an earlier stage, it is critical to work on improving the yield rates for biopsy of smaller lesions. Further studies are needed to determine the best combination of adjunct imaging and sampling modality to capture the greatest yield in this category.

In summary, our analysis of 1084 robotic bronchoscopic cases adopted by multiple users across pulmonology and thoracic surgery demonstrates that this technology achieves a high diagnostic success (82.6%) with a low complication rate. Diagnostic performance improves further when lesions are selected for size and imaging characteristics. These findings support the use of robotic bronchoscopy as a preferred approach for the biopsy of suspicious pulmonary lesions.

## Supplementary Material

Supplemental Tables

Supplementary Figure 1

Supplementary data related to this article can be found at https://doi.org/10.1016/j.jss.2026.04.031.

## Figures and Tables

**Table 1 –:** Operative data.

Characteristic	Total, *N*= 1022

Specialty of operator, *N* (%)	
Pulmonology	690, 67.5%
Thoracic surgery	332, 32.5%
Case time, min, median (IQR)	58 (46–73)
Case type, *N* (%)	
Inpatient	93, 9.1%
Outpatient	929, 90.9%
Repeat monarch	46, 4.5%
Number of lesions biopsied	
1	965, 94.4%
2	54, 5.3%
3	3, 0.3%
Use of fluoroscopy, *N* (%)	986, 96.6%
Traditional EBUS, *N* (%)	547, 53.5%
Additional procedures, *N*	48
Esophagogastroduodenoscopy	25
Chemotherapy port placement	10
Fiducial marker placement	6

**Table 2 –:** Patient data.

Characteristic	Total, *N*= 1022

Age, median (IQR)	68.9 (62.90–75.58)
Sex, No (%)	
Female	546 (53.4)
Male	476 (46.6)
Race, no (%)	
White	945 (92.5)
Black	61 (6.0)
Asian	6 (0.6)
Other	10 (1.0)
BMI, kgm2, median (IQR)	26.91 (22.97–36.67)
Smoking status, no (%)	
Current	364 (35.6)
Former	527 (51.6)
Never	13 (12.8)
History of COPD	537 (52.5)
History of lung cancer	113 (11.1)
History of other cancer	322 (31.5)
History of chest surgery	163 (16.0)
ECOG 0–1	592 (84.8)

**Table 3 –:** Univariate comparison of patient and surgical characteristics and outcomes based on diagnostic success.

Characteristic	Diagnostic success		
	
	No	Yes	*P* value*
			
	*N* = 150	*N* = 715	

Age (years), Median (IQR)	70 (63–76)	69 (63–75)	0.79
Sex, *n* (%)			0.16
Female	90 (60)	384 (54)	
Male	60 (40)	331 (46)	
Race, *n* (%)			0.67
Black	10 (6.7)	41 (5.7)	
Asian	0 (0)	5 (0.7)	
White	138 (92)	663 (93)	
Other	1 (0.7)	3 (0.4)	
Unknown	1 (0.7)	3 (0.4)	
Weight (kg), median (IQR)	79 (65–90)	77 (64–91)	0.27
Body mass index (kg/m^2^), median (IQR)	28 (24–33)	27 (23–32)	0.055
Body mass index (kg/m^2^), *n* (%)			0.14
< 30.0	92 (61)	492 (69)	
30.0–34.9	37 (25)	129 (18)	
35+	21 (14)	94 (13)	
Smoking status, *n* (%)			0.50
Current	57 (38)	248 (35)	
Former	77 (51)	367 (51)	
Never	16 (11)	100 (14)	
History of COPD, *n* (%)	74 (49)	370 (52)	0.59
History of chest surgery, *n* (%)	27 (18)	114 (16)	0.55
ECOG 0/1, *n* (%)	104 (97)	403 (94)	0.23
Lung, *n* (%)			0.053
Left	54 (36)	319 (45)	
Right	96 (64)	396 (55)	
Long axis, median (IQR)	21 (15–30)	27 (18–40)	<0.001
Short axis, median (IQR)	15 (12–22)	20 (14–30)	<0.001
Nodule region, *n* (%)			0.003
Central	43 (29)	299 (42)	
Peripheral = beyond lobar bronchus	107 (71)	416 (58)	
Pleural distance, median (IQR)	10 (0–22)	6 (0–19)	0.015
Nodule character, *n* (%)			0.49
GGO	4 (2.7)	16 (2.2)	
Mixed	23 (15)	88 (12)	
Solid	122 (82)	608 (85)	
Bronchus sign, *n* (%)	85 (57)	533 (75)	<0.001
Case type, *n* (%)			0.002
Inpatient	3 (2.0)	70 (9.8)	
Outpatient	146 (98)	641 (90)	
Repeat Monarch, *n* (%)	10 (6.7)	26 (3.6)	0.093
Number of lesions biopsied, *n* (%)			0.90
1	138 (92)	649 (91)	
2	12 (8.0)	61 (8.5)	
3	0 (0)	5 (0.7)	
Use of fluoroscopy, *n* (%)	145 (97)	692 (97)	0.80
Traditional EBUS, *n* (%)	58 (39)	400 (56)	<0.001
R-EBUS localized, *n* (%)	77 (51)	509 (71)	<0.001
Lesion visible on endoscope, *n* (%)	20 (14)	181 (26)	0.002
Distance from scope to lesion, median (IQR)	15 (10–20)	15 (10–20)	0.091

* Pearson’s chi-squared test; Wilcoxon rank sum test; Fisher’s exact test; Fisher’s exact test for count data with simulated *P* value (based on 2000 replicates).

**Table 4 –:** Monarch success by tumor size and nodule characteristic.

Characteristic	Monarch success		
	
	No *N* = 149	Yes *N* = 712	*P* value*

Solid nodule type (*n* = 730)			
Long axis size, *n* (%)			<0.001
< 1 cm	1 (11)	8 (89)	
1–2 cm	59 (26)^†^	172 (74)^†^	
2–3 cm	33 (16)	171 (84)	
3+ cm	29 (11)^†^	241 (89)^†^	
Mixed nodule type (*n* = 111)			
Long axis size, *n* (%)			0.19
1–2 cm	7 (30)	16 (70)	
2–3 cm	8 (24)	26 (76)	
3+ cm	7 (13)	45 (87)	
GGO nodule type (*n* = 20)			
Long axis size, *n* (%)			0.76
1–2 cm	3 (30)	7 (70)	
2–3 cm	1 (25)	3 (75)	
3+ cm	0 (0)	4 (100)	

* Fisher’s exact test.

† *P* < 0.05 post hoc.

**Table 5 –:** Multivariable prediction of successful Monarch diagnosis.

Characteristic	Odds ratio (95% CI)	*P* value

Body mass index	0.99 (0.99–1.00)	0.003
Repeat monarch		
No	–	
Yes	0.86 (0.76–0.98)	0.022
Laterality		
Left	–	
Right	0.94 (0.89–0.99)	0.029
Short axis (cm)	1.00 (1.00–1.01)	0.008
Bronchus sign		
No	–	
Yes	1.14 (1.07–1.22)	<0.001
R-EBUS localized		
No	–	
Yes	1.20 (1.12–1.28)	<0.001

CI = confidence interval.

**Table 6 –:** Comparison of patient and surgical characteristics between Monarch indeterminate, Monarch benign (successful), andMonarch malignant (successful) diagnoses.

Characteristic	Overall, *N* = 816	Benign, *N* = 244	Indeterminate, *N* = 102	Malignant, *N* = 470	*P* value*

Age, median (IQR)	69 (63–75)	67 (61–74)^†^	67 (60–75)^†^	70 (64–76)^†^	<0.001
Sex, *n* (%)					0.35
Female	434 (53)	124 (51)	50 (49)	260 (55)	
Male	382 (47)	120 (49)	52 (51)	210 (45)	
Height (cm), median (IQR)	168 (160–175)	169 (160–178)	168 (163–175)	168 (160–175)	0.46
Weight (kg), median (IQR)	78 (64–92)	78 (61–93)	80 (65–93)	77 (65–91)	0.57
Body mass index (kg/m^2^), median (IQR)	27 (23–32)	26 (22–31)	28 (23–33)	27 (23–32)	0.28
Smoking history, *n* (%)					<0.001
Current	291 (36)	81 (33)	43 (42)	167 (36)	
Former	421 (52)	110 (45)^†^	55 (54)	256 (54)	
Never	104 (13)	53 (22)^†^	4 (3.9)^†^	47 (10)^†^	
COPD, *n* (%)	440 (54)	127 (52)	70 (69)^†^	243 (52)	0.006
Asthma, *n* (%)	127 (16)	50 (20)^†^	15 (15)	62 (13)^†^	0.038
ILD, *n* (%)	14 (1.7)	7 (2.9)	3 (2.9)	4 (0.9)	0.061
Lung cancer history, *n* (%)	82 (10)	21 (8.6)	14 (14)	47 (10)	0.35
Lung cancer family history, *n* (%)	176 (22)	57 (23)	21 (21)	98 (21)	0.70
Occupational exposure, *n* (%)	76 (9.4)	33 (14)^†^	7 (6.9)	36 (7.8)	0.028
Previous chest surgery, *n* (%)	126 (16)	35 (14)	13 (13)	78 (17)	0.51
RAI, median (IQR)	32 (24–37)	27 (21–35)^†^	33 (24–37)^†^	34 (25–38)^†^	0.001
Charlson comorbidity Index, median (IQR)	1.00 (0.00–3.00)	1.00 (0.00–3.00)^†^	2.00 (1.00–3.00)^†^	1.00 (0.00–3.00)^†^	0.022
Other cancer history, *n* (%)	242 (30)	67 (27)	34 (33)	141 (30)	0.53
Lung, *n* (%)					0.24
Left	355 (44)	105 (43)	37 (36)	213 (45)	
Right	461 (56)	139 (57)	65 (64)	257 (55)	
Long axis, median (IQR)	26 (18–39)	24 (16–36)^†^	23 (16–36)^†^	29 (20–42)^†^	<0.001
Short axis, median (IQR)	20 (13–30)	17 (12–25)^†^	17 (12–29)^†^	21 (15–32)^†^	<0.001
Nodule region, *n* (%)					<0.001
Central	334 (41)	72 (30)^†^	34 (33)	228 (49)^†^	
Peripheral = beyond lobar bronchus	482 (59)	172 (70)^†^	68 (67)	242 (51)^†^	
Pleural distance, median (IQR)	6 (0–19)	3 (0–17)	5 (0–20)	7 (0–19)	0.20
Cavitary, *n* (%)	95 (12)	41 (17)^†^	12 (12)	42 (8.9)^†^	0.008
Solid, *n* (%)	687 (84)	197 (81)	79 (77)^†^	411 (87)^†^	0.009
Spiculated, *n* (%)	366 (45)	94 (39)	46 (45)	226 (48)	0.051
GGO, *n* (%)	19 (2.3)	8 (3.3)	3 (2.9)	8 (1.7)	0.33
Mixed, *n* (%)	107 (13)	38 (16)	20 (20)†	49 (10)^†^	0.018
Lobulated, *n* (%)	68 (8.3)	15 (6.1)	8 (7.8)	45 (9.6)	0.29
Necrotic, *n* (%)	36 (4.4)	9 (3.7)	1 (1.0)	26 (5.6)	0.10
Bronchus sign, *n* (%)	597 (73)	162 (66)^†^	65 (64)^†^	370 (79)^†^	<0.001
R-EBUS localized, *n* (%)	573 (70)	157 (64)^†^	67 (66)	349 (74)^†^	0.013
R-EBUS image, *n* (%)					<0.001
Concentric	372 (71)	86 (61)^†^	31 (53)^†^	255 (78)^†^	
Eccentric	155 (29)	56 (39)^†^	28 (47)^†^	71 (22)^†^	
Lesion visible on endoscopy, *n* (%)	200 (25)	39 (16)^†^	20 (20)	141 (30)^†^	<0.001
PET avid, *n* (%)	543 (89)	102 (82)^†^	67 (86)	374 (92)^†^	0.009
Number of lesions biopsied, *n* (%)					0.006
1	744 (91)	212 (87)^†^	96 (94)	436 (93)	
2	67 (8.2)	27 (11)	6 (5.9)	34 (7.2)	
3	5 (0.6)	5 (2.0)^†^	0 (0)	0 (0)^†^	
Postprocedure pneumothorax, *n* (%)	34 (4.2)	10 (4.1)	5 (4.9)	19 (4.1)	0.91
Postoperative chest tube, *n* (%)	22 (35)	7 (37)	4 (44)	11 (31)	0.76
Postoperative other complication, *n* (%)	21 (2.6)	2 (0.8)	3 (2.9)	16 (3.4)	0.086
Lobe, *n* (%)					0.17
Left lower	114 (14)	38 (16)	18 (18)	58 (12)	
Left lingula	46 (5.6)	13 (5.3)	5 (4.9)	28 (6.0)	
Left upper	195 (24)	54 (22)	14 (14)^†^	127 (27)^†^	
Right lower	141 (17)	43 (18)	18 (18)	80 (17)	
Right middle	64 (7.8)	16 (6.6)	7 (6.9)	41 (8.7)	
Right upper	256 (31)	80 (33)	40 (39)	136 (29)	
Segment category, *n* (%)					0.003
Left lower anteromedial basal (S7/S8)	14 (1.7)	6 (2.5)	2 (2.0)	6 (1.3)	
Left lingula inferior (S5)	22 (2.7)	5 (2.0)	4 (3.9)	13 (2.8)	
Left lower lateral basal (S9)	16 (2.0)	7 (2.9)	3 (2.9)	6 (1.3)	
Left lower superior (S6)	49 (6.0)	15 (6.1)	10 (9.8)	24 (5.1)	
Left lower posterior basal (S10)	35 (4.3)	10 (4.1)	3 (2.9)	22 (4.7)	
Left lingula superior (S4)	24 (2.9)	8 (3.3)	1 (1.0)	15 (3.2)	
Left upper anterior (S3)	62 (7.6)	15 (6.1)	3 (2.9)	44 (9.4)	
Left upper apicoposterior (S1/S2)	133 (16)	39 (16)	11 (11)	83 (18)	
Right lower anterior basal (S8)	24 (2.9)	8 (3.3)	4 (3.9)	12 (2.6)	
Right lower lateral basal (S9)	22 (2.7)	8 (3.3)	2 (2.0)	12 (2.6)	
Right lower medial basal (S7)	8 (1.0)	3 (1.2)	1 (1.0)	4 (0.9)	
Right lower superior (S6)	53 (6.5)	17 (7.0)	5 (4.9)	31 (6.6)	
Right lower posterior basal (S10)	34 (4.2)	7 (2.9)	6 (5.9)	21 (4.5)	
Right middle lateral (S4)	38 (4.7)	9 (3.7)	6 (5.9)	23 (4.9)	
Right middle medial (S5)	26 (3.2)	7 (2.9)	1 (1.0)	18 (3.8)	
Right upper anterior (S3)	70 (8.6)	14 (5.7)	2 (2.0)	54 (11)^†^	
Right upper apical (S1)	104 (13)	31 (13)	26 (25) ^†^	47 (10)^†^	
Right upper posterior (S2)	82 (10)	35 (14)^†^	12 (12)	35 (7.4)^†^	

* Pearson’s Chi-squared test; Kruskal–Wallis rank sum test; Fisher’s exact test; Fisher’s exact test for count data with simulated *P* value (based on 2000 replicates).

† *P* < 0.05 post hoc.
