# Supplementary material for: Diagnostic Outcomes of Robotic Bronchoscopy in a Multidisciplinary, Multi-hospital Lung Nodule Program
Source: J Surg Res. Author manuscript; Available in PMC 2026 Sep 14. (PMC13574333; doi:10.1016/j.jss.2026.04.031)
Supplement: Supplementary file 1 [file NIHMS2201738-supplement-Supplemental_Tables.docx]

Supplemental Tables

Supplemental Table 1. Nodule Characteristics

| **Characteristic** | **Total, N= 1084** |
| --- | --- |
| Lung, n (%) |  |
| Left | 457 (42) |
| Right | 627 (58) |
| Segment, n (%) |  |
| Left Lower Anteromedial Basal (S7/S8) | 21 (1.9) |
| Left Lingula Inferior (S5) | 27 (2.5) |
| Left Lower Lateral Basal (S9) | 26 (2.4) |
| Left Lower Superior (S6) | 57 (5.3) |
| Left Lower Posterior Basal (S10) | 46 (4.2) |
| Left Lingula Superior (S4) | 33 (3.0) |
| Left Upper Anterior (S3) | 79 (7.3) |
| Left Upper Apicoposterior (S1/S2) | 168 (15) |
| Right Lower Anterior Basal (S8) | 32 (3.0) |
| Right Lower Lateral Basal (S9) | 33 (3.0) |
| Right Lower Medial Basal (S7) | 11 (1.0) |
| Right Lower Superior (S6) | 71 (6.5) |
| Right Lower Posterior Basal (S10) | 50 (4.6) |
| Right Middle Lateral (S4) | 44 (4.1) |
| Right Middle Medial (S5) | 33 (3.0) |
| Right Upper Anterior (S3) | 98 (9.0) |
| Right Upper Apical (S1) | 136 (13) |
| Right Upper Posterior (S2) | 119 (11) |
| Long Axis, Median (IQR) | 25 (17 – 37) |
| Short Axis, Median (IQR) | 18 (13 – 27) |
| Nodule Region, n (%) |  |
| Central | 417 (38) |
| Peripheral = beyond lobar bronchus | 667 (62) |
| Pleural Distance, Median (IQR) | 7 (0 – 20) |
| Nodule Character, n (%) |  |
| GGO | 30 (2.8) |
| Mixed | 148 (14) |
| Solid | 901 (84) |
| Bronchus Sign, n (%) | 760 (70) |
| Rebus Localized, n (%) | 713 (66) |
| Lesion Visible on Endoscope, n (%) | 230 (22) |

Supplemental Table 2. Univariable Comparison of Demographic and Surgical Comparison Based on Lesion Reached.

|  | **Lesion Reached** | |  |
| --- | --- | --- | --- |
| **Characteristic** | **Yes**  N = 1,084 | **No**  N = 29 | **p-value**^1^ |
| Case Time (min), Median (IQR) | 59 (46 – 75) | 69 (59 – 96) | 0.007 |
| Case Type, n (%) |  |  | 0.17 |
| Inpatient | 96 (8.9) | 0 (0) |  |
| Outpatient | 983 (91) | 29 (100) |  |
| Repeat Monarch, n (%) | 46 (4.3) | 1 (3.6) | >0.99 |
| Number of Lesions Biopsied, n (%) |  |  | <0.001 |
| 0 | 0 (0) | 13 (45) |  |
| 1 | 969 (89) | 15 (52) |  |
| 2 | 106 (9.8) | 1 (3.4) |  |
| 3 | 9 (0.8) | 0 (0) |  |
| Use of Fluoroscopy, n (%) | 1,045 (96) | 23 (79) | <0.001 |
| Traditional EBUS, n (%) | 564 (52) | 17 (59) | 0.48 |
| Age (years), Median (IQR) | 69 (63 – 76) | 74 (63 – 80) | 0.082 |
| Sex, n (%) |  |  | 0.88 |
| Female | 583 (54) | 16 (55) |  |
| Male | 501 (46) | 13 (45) |  |
| Race, n (%) |  |  | 0.20 |
| Black | 63 (5.8) | 0 (0) |  |
| Asian | 6 (0.6) | 1 (3.4) |  |
| White | 1,005 (93) | 28 (97) |  |
| Other | 5 (0.5) | 0 (0) |  |
| Unknown | 5 (0.5) | 0 (0) |  |
| Weight (kg), Median (IQR) | 77 (64 – 91) | 72 (64 – 90) | 0.44 |
| Body Mass Index (kg/m²), Median (IQR) | 27 (23 – 32) | 25 (22 – 29) | 0.25 |
| Smoking Status, n (%) |  |  | 0.70 |
| Current | 389 (36) | 9 (31) |  |
| Former | 556 (51) | 15 (52) |  |
| Never | 139 (13) | 5 (17) |  |
| History of Pulmonary Hypertension, n (%) | 39 (3.6) | 2 (6.9) | 0.29 |
| History of COPD, n (%) | 568 (52) | 16 (55) | 0.77 |
| History of Lung Cancer, n (%) | 119 (11) | 6 (21) | 0.13 |
| History of Other Cancer, n (%) | 344 (32) | 11 (38) | 0.48 |
| History of Chest Surgery, n (%) | 171 (16) | 6 (21) | 0.44 |
| ECOG 0/1, n (%) | 627 (95) | 24 (100) | 0.62 |
| Lung, n (%) |  |  | 0.12 |
| Left | 457 (42) | 8 (28) |  |
| Right | 627 (58) | 21 (72) |  |
| Lobe, n (%) |  |  | 0.64 |
| Lingula | 60 (5.5) | 0 (0) |  |
| Lower | 347 (32) | 9 (31) |  |
| Middle | 77 (7.1) | 1 (3.4) |  |
| Upper | 600 (55) | 19 (66) |  |
| Lobe Category, n (%) |  |  | 0.54 |
| Left Lower | 150 (14) | 2 (6.9) |  |
| Left Lingula | 60 (5.5) | 0 (0) |  |
| Left Upper | 247 (23) | 6 (21) |  |
| Right Lower | 197 (18) | 7 (24) |  |
| Right Middle | 77 (7.1) | 1 (3.4) |  |
| Right Upper | 353 (33) | 13 (45) |  |
| Long Axis, Median (IQR) | 25 (17 – 37) | 16 (13 – 27) | 0.002 |
| Short Axis, Median (IQR) | 18 (13 – 27) | 13 (10 – 19) | <0.001 |
| Nodule Region, n (%) |  |  | 0.49 |
| Central | 417 (38) | 13 (45) |  |
| Peripheral = beyond lobar bronchus | 667 (62) | 16 (55) |  |
| Pleural Distance, Median (IQR) | 7 (0 – 20) | 6 (0 – 14) | 0.72 |
| Nodule Character, n (%) |  |  | 0.74 |
| GGO | 30 (2.8) | 0 (0) |  |
| Mixed | 148 (14) | 5 (17) |  |
| Solid | 901 (84) | 24 (83) |  |
| Bronchus Sign, n (%) | 760 (70) | 12 (41) | <0.001 |
| R-EBUS Localized, n (%) | 713 (66) | 5 (18) | <0.001 |
| Lesion Visible on Endoscope, n (%) | 230 (22) | 0 (0) | 0.006 |
| Distance from Scope to Lesion, Median (IQR) | 15 (10 – 20) | 17 (10 – 50) | 0.47 |
| ^1^Pearson's Chi-squared test; Wilcoxon rank sum test; Fisher's exact test; Fisher's Exact Test for Count Data with simulated p-value (based on 2000 replicates) | | | |

***Supplemental Table 3****. Post-operative Complications*

| **Complication** | **Frequency, N** | **Admission, N** |
| --- | --- | --- |
| Hypoxic respiratory failure, requiring admission (no intubation required) | 8 | 8 |
| New O2 requirement post-op, discharged on oxygen per nasal cannula | 7 | 7 |
| Brief episode of VT at termination of procedure | 1 | 1 |
| New onset atrial fibrillation with rapid ventricular response (RVR) | 1 | 1 |
| Cardiopulmonary collapse upon initiation of procedure, pulseless electrical activity (PEA) arrest and death | 1 | 0 |
| ED visit for bronchospasm on POD 3 | 1 | 0 |
| Acute status epilepticus with acute metabolic encephalopathy | 1 | 1 |
| Admission through ED on POD 1 for weakness and confusion | 1 | 1 |
| Admission through ED on POD 0 for STEMI | 1 | 1 |
| Intubation for AMS post-procedurally | 1 | 1 |
| Post-op CXR opacification of hemithorax requiring therapeutic bronchoscopy | 1 | 0 |
| Endobronchial bleeding, remained intubated post-operatively | 3 | 3 |
| Pneumothorax, not requiring chest tube | 18 | 7 |
| Pneumothorax, requiring tube thoracostomy | 30 | 30 |
| Delayed presentation of pneumothorax (POD 2-POD9) | 3 | 3 |

***Supplemental Table*** *4. Characteristics Associated with Tube Thoracostomy*

|  | | **Thoracostomy** | |  |
| --- | --- | --- | --- | --- |
| **Characteristic** | **N** | **No**  N = 1,051 | **Yes**  N = 33 | **p-value**^1^ |
| Age (years), Median (IQR) | 1,084 | 69 (63 – 76) | 68 (61 – 73) | 0.21 |
| Sex, n (%) | 1,084 |  |  | 0.93 |
| Female |  | 565 (54) | 18 (55) |  |
| Male |  | 486 (46) | 15 (45) |  |
| Race, n (%) | 1,084 |  |  | 0.55 |
| Black |  | 63 (6.0) | 0 (0) |  |
| Asian |  | 6 (0.6) | 0 (0) |  |
| White |  | 972 (92) | 33 (100) |  |
| Other |  | 5 (0.5) | 0 (0) |  |
| Unknown |  | 5 (0.5) | 0 (0) |  |
| Weight (kg), Median (IQR) | 1,084 | 77 (64 – 91) | 75 (65 – 89) | 0.64 |
| Body Mass Index (kg/m²), Median (IQR) | 1,084 | 27 (23 – 32) | 26 (23 – 31) | 0.58 |
| Smoking Status, n (%) | 1,084 |  |  | 0.64 |
| Current |  | 378 (36) | 11 (33) |  |
| Former |  | 540 (51) | 16 (48) |  |
| Never |  | 133 (13) | 6 (18) |  |
| History of Pulmonary Hypertension, n (%) | 1,082 | 38 (3.6) | 1 (3.1) | >0.99 |
| History of COPD, n (%) | 1,084 | 550 (52) | 18 (55) | 0.80 |
| History of Lung Cancer, n (%) | 1,084 | 115 (11) | 4 (12) | 0.78 |
| History of Other Cancer, n (%) | 1,084 | 331 (31) | 13 (39) | 0.34 |
| History of Occupational Exposure, n (%) | 1,077 | 93 (8.9) | 3 (9.1) | >0.99 |
| History of Chest Surgery, n (%) | 1,080 | 167 (16) | 4 (12) | 0.55 |
| ECOG 0/1, n (%) | 659 | 610 (95) | 17 (94) | 0.60 |
| Lung, n (%) | 1,084 |  |  | 0.97 |
| Left |  | 443 (42) | 14 (42) |  |
| Right |  | 608 (58) | 19 (58) |  |
| Lobe, n (%) | 1,084 |  |  | 0.052 |
| Lingula |  | 57 (5.4) | 3 (9.1) |  |
| Lower |  | 337 (32) | 10 (30) |  |
| Middle |  | 71 (6.8) | 6 (18) |  |
| Upper |  | 586 (56) | 14 (42) |  |
| Lobe Category, n (%) | 1,084 |  |  | 0.16 |
| Left Lower |  | 146 (14) | 4 (12) |  |
| Left Lingula |  | 57 (5.4) | 3 (9.1) |  |
| Left Upper |  | 240 (23) | 7 (21) |  |
| Right Lower |  | 191 (18) | 6 (18) |  |
| Right Middle |  | 71 (6.8) | 6 (18) |  |
| Right Upper |  | 346 (33) | 7 (21) |  |
| Segment Category, n (%) | 1,084 |  |  | 0.063 |
| Left Lower Anteromedial Basal (S7/S8) |  | 19 (1.8) | 2 (6.1) |  |
| Left Lingula Inferior (S5) |  | 25 (2.4) | 2 (6.1) |  |
| Left Lower Lateral Basal (S9) |  | 25 (2.4) | 1 (3.0) |  |
| Left Lower Superior (S6) |  | 56 (5.3) | 1 (3.0) |  |
| Left Lower Posterior Basal (S10) |  | 46 (4.4) | 0 (0) |  |
| Left Lingula Superior (S4) |  | 32 (3.0) | 1 (3.0) |  |
| Left Upper Anterior (S3) |  | 76 (7.2) | 3 (9.1) |  |
| Left Upper Apicoposterior (S1/S2) |  | 164 (16) | 4 (12) |  |
| Right Lower Anterior Basal (S8) |  | 32 (3.0) | 0 (0) |  |
| Right Lower Lateral Basal (S9) |  | 30 (2.9) | 3 (9.1) |  |
| Right Lower Medial Basal (S7) |  | 10 (1.0) | 1 (3.0) |  |
| Right Lower Superior (S6) |  | 70 (6.7) | 1 (3.0) |  |
| Right Lower Posterior Basal (S10) |  | 49 (4.7) | 1 (3.0) |  |
| Right Middle Lateral (S4) |  | 42 (4.0) | 2 (6.1) |  |
| Right Middle Medial (S5) |  | 29 (2.8) | 4 (12) |  |
| Right Upper Anterior (S3) |  | 95 (9.0) | 3 (9.1) |  |
| Right Upper Apical (S1) |  | 134 (13) | 2 (6.1) |  |
| Right Upper Posterior (S2) |  | 117 (11) | 2 (6.1) |  |
| Long Axis, Median (IQR) | 1,084 | 25 (17 – 37) | 25 (18 – 35) | 0.93 |
| Short Axis, Median (IQR) | 1,084 | 18 (13 – 27) | 18 (12 – 24) | 0.49 |
| Nodule Region, n (%) | 1,084 |  |  | 0.91 |
| Central |  | 404 (38) | 13 (39) |  |
| Peripheral/Beyond Lobar Bronchus |  | 647 (62) | 20 (61) |  |
| Pleural Distance, Median (IQR) | 1,084 | 7 (0 – 20) | 6 (0 – 23) | 0.88 |
| Nodule Character, n (%) | 1,079 |  |  | 0.12 |
| GGO |  | 28 (2.7) | 2 (6.1) |  |
| Mixed |  | 141 (13) | 7 (21) |  |
| Solid |  | 877 (84) | 24 (73) |  |
| Bronchus Sign, n (%) | 1,084 | 741 (71) | 19 (58) | 0.11 |
| R-EBUS Localized, n (%) | 1,083 | 698 (66) | 15 (45) | 0.012 |
| Lesion Visible on Endoscope, n (%) | 1,066 | 226 (22) | 4 (12) | 0.18 |
| Distance from Scope to Lesion, Median (IQR) | 866 | 15 (10 – 20) | 17 (11 – 30) | 0.13 |
| ^1^Wilcoxon rank sum test; Pearson's Chi-squared test; Fisher's exact test; Fisher's Exact Test for Count Data with simulated p-value (based on 2000 replicates)  * p < 0.05 post hoc | | | | |

***Supplemental Table 5****. Final Malignant Pathology*

| **Characteristic** | **Total N** |
| --- | --- |
| Final Pathology |  |
| Adenocarcinoma | 212 |
| Squamous Cell Carcinoma | 134 |
| Metastasized Cancer | 34 |
| Small Cell Carcinoma | 24 |
| NSCLC, NOS | 20 |
| Other | 46 |
| Surgical Resection Performed | 261 |
| Tumor T Stage |  |
| T1a | 2 |
| T1b | 68 |
| T1c | 44 |
| T2a | 29 |
| T2b | 25 |
| T3 | 23 |
| T4 | 11 |
| SBRT | 121 |
| Ablation | 4 |
| Chemotherapy, Immunotherapy | 320 |
| Radiation | 140 |

**Supplemental Figure Legend**

Supplemental Figure 1. Post-biopsy treatment of indeterminate lesions
